# Revival of the side-to-side approach for distal coronary anastomosis

**DOI:** 10.1186/1749-8090-2-2

**Published:** 2007-01-06

**Authors:** Min-Ho Song, Yoshiyuki Tokuda, Toshiaki Ito

**Affiliations:** 1 Department of Cardiovascular Surgery, Gifu Prefectural Tajimi Hospital, Tajimi, Gifu, Japan; 2 Department of Cardiovascular Surgery, The Japanese Red Cross Nagoya First Hospital, Nagoya, Japan

## Abstract

Side-to-side anastomosis was employed by just ten proportional stitches while performing distal anastomosis during coronary artery surgery. This technique is simple and quick. Here this simple technique is described in detail and the postoperative status of grafted conduits is reported.

## Background

As new devices for automated anastomosis are developed, hand-sewn anastomosis appears to be on the wane. As for proximal anastomosis during coronary artery bypass grafting, several aortic connecting devices have some favorable prospects although their safety has not been yet been proven [1]. In addition, regarding distal anastomosis, new anastomosing devices, such as the retinol interrupted anastomosis device and magnetic device, have been developed [2], with some favorable prospects. As surgeons who take pride in craftsmanship and proficiency, we have been working on our skill and employ a technique of side-to-side anastomosis with just ten proportional stitches by hand during coronary surgery.

**Figure 1:**
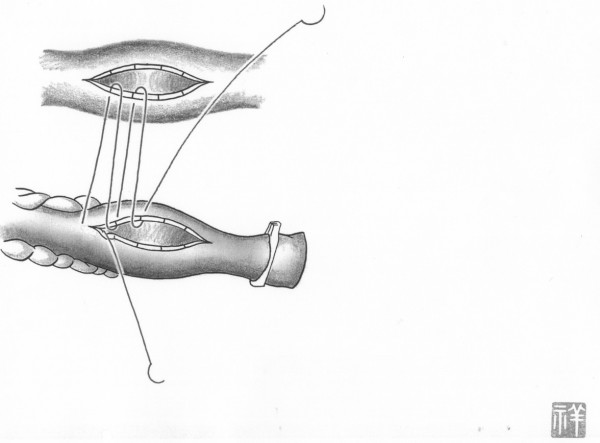
**Initial Anastomosis from the Surgeon's Viewpoint**. The original coronary artery is shown at the top and the graft artery is shown at the bottom. In this technique, the graft artery is placed near the surgeon. This suturing technique begins at the point two-fifths halfway towards the arteriotomy near heel position. The suture is made from outside of the graft to inside of the graft. Consequently the next suture is made at the corresponding point of coronary artery from inside of coronary artery to outside of coronary artery. The same forehand continues to place stitches in a clockwise rotation. Black and white segments are hypothetical segments and they correspond to the one-tenth division of anastomosing orifice. Consecutively single-parachute stitches are done up to the fifth point.

**Figure 2:**
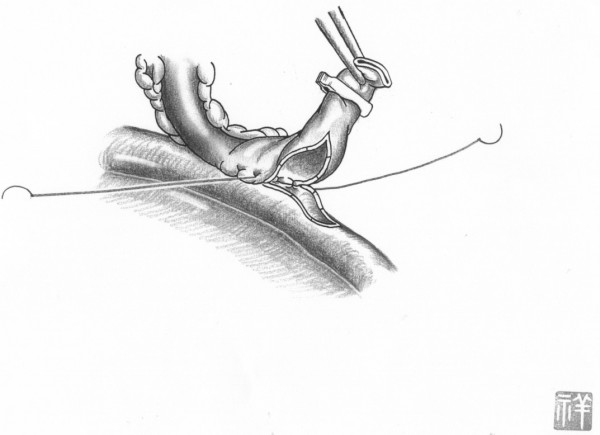
**Completing anastomosis from the Surgeon's Viewpoint**. The graft conduit is then pulled down to the original coronary and the surgeon confirms that the stitches are securely pulled down. Without changing needle holder, the next stitch is put at the sixth point in a backhand manner from the outside of the graft, and inside of both the graft and coronary artery, and then outside of the coronary artery in one action. Stitching continues at the seventh, the eighth (just the toe position), the ninth, and finally the tenth point.

**Figure 3:**
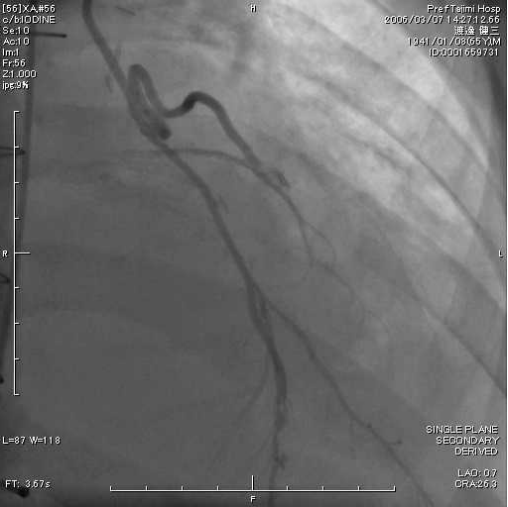
**Typical Postoperative Angiogram**. In this case, a Y composite was made by attaching the radial artery to the left internal mammary artery, shown from the left anterior descending and an obtuse marginal anastomoses is wide open.

In both on-pump arrested heart and off-pump beating heart, in situ the conduit artery (mammary or gastroepiploic) is put along side the target coronary artery at about twenty millimeters. The conduit is put near the operator in most cases. After making the appropriate arteriotomy in length (usually one and half times longer than the coronary diameter), in accordance with the results obtained from computational fluid dynamics in both conduits and native coronary [3], the first suture is made at a point two-fifths the length of the conduit from the outside towards the inside of the conduit. By weighting a light bulldog clamp onto the other arm of the suture, the anastomosing orifice of conduit becomes wide and clear. Forehand suturing continues at the point two-fifths the length of the native coronary, doing so inside to the outside of the native coronary (Figure 1). The conduit is pulled down after making five stitches around the heel position of the coronary artery. This suturing continues in a clockwise direction and is completed by backhand suturing and forehand suturing after making a turnaround at the toe position (Figure 2). Figure 3 shows the typical postoperative angiogram of a Y composite graft made by attaching the radial artery to the left internal mammary artery and are wide open.

From April 2003 to March 2004, 186 distal anastomoses were performed by this technique; left internal mammary artery was used in 93 anastomoses, right internal mammary artery was used in 21, right gastroepiploic artery was used in 37, and radial artery was used in 35. 52 anastomoses were performed off-pump and the remaining 134 anastomoses were performed on-pump. We exclude vein grafts in this study and always used in-situ arterial grafts to avoid aortic manipulationin in off-pump cases. The overall patency rate of the anastomosis was 100%, and was usually confirmed by postoperative coronary angiography on the 10^th ^postopearitve day. Contrary to general fears and briefings, a clogged up end of a graft did not affect anastomosis. The average time for completing one anastomsis was 6 minutes and 17 seconds (standard deviation was 69 seconds). Surprisingly, there was only three anastomosis requiring additional sutures to stop bleeding from anastomosis through this series. All grafts were examined, scrutinizing the functioning status by means of intraoperative transit Doppler flow meter (Medistim, Oslo, Norway) after termination of cardiopulmonary bypass and reversal of heparin. Good functioning arterial grafts could be determined by ascertaining the diastolic dominant flow pattern of the flow, perfusion index less than 5.0, and a flow more than 10 ml/min. In this series, the average flow was as follows; left internal mammary artery 39 +/- 11 ml/min, right internal mammary artery 43 +/- 4 ml/min, right gastroepiploic artery 18 +/- 10 ml/min, and radial artery 28 +/- 13 ml/min. Two grafts were judged to be mal-functioning as no diastolic component was shown on the Doppler. The distal ends of these anastomoses were opened by removing the clips and the anastomoses were examined by direct vision. We passed a 1.0 mm or 1.5 mm probe to make sure that they were wide open.

The two grafts were found to be vasospastic and were treated by a topical spray of warm milrinone solution.

This technique has not yet been utilized in on-pump cases needing vein grafts, because clogging of vein grafts using traditional end-to-side anastomosis has not yet occurred. We are now working on an evaluation of vein graft status using this technique.

Side-to-side anastomosis has four advantages [4]. First, inserting proportional sutures is easy and is not misleading. The coronary incision and graft incision can be perfectly matched by applying ten proportional stitches. Second, it is easy to confirm anastomosis if there is a doubt. All that is necessary is to reopen the anastomosis via the distal end of the graft. An anastomosis need not be re-sutured. Third, the distal end of the graft can be held beyond the surgical clip by forceps without damaging the arterial graft, making it easier to perform anastomosis [4]. Fourth, side-to-side anastomosis is superior to end-to-side-anastomosis in the light of fluid dynamics [5]. Bonert, et al., from Toronto stated that a parallel configuration has fewer areas of low wall shear stress and low spatial wall shear stress gradients, and therefore is preferred over the diamond for maintaining graft patency.

We conclude that this technique is attractive and straightforward because it needs only seven minutes to finish, gives direct vision of anastomosis in the event of a problem without the need to repeat sutures, and gives relief in terms of superior fluid dynamics in the blood flow of anastomosis when completing an anastomosis.

## References

[B1] Vicol C, Oberhoffer M, Nollert G, Eifert S, Boekstegers P, Wintersperger B, Reichart B (2005). First clinical experience with the HEARTSTRING, a device for proximal anastomoses in coronary surgery. Ann Thorac Surg.

[B2] Filsoufi F, Farivar RS, Aklog L, Anderson CA, Chen RH, Lichtenstein S, Zhang J, Adams DH (2004). Automated distal coronary bypass with a novel magnetic coupler (MVP system). J Thorac Cardiovasc Surg.

[B3] Song MH, Sato M, Ueda Y (2000). Three-dimensional simulation of coronary artery bypass grafting with the use of computational fluid dynamics. Surg Today.

[B4] Niinami H, Takeuchi Y (2000). Coronary artery bypass grafting using side-to-side anastomosis. Kyobu Geka.

[B5] Bonert M, Myers JG, Fremes S, Williams J, Ethier CR (2002). A numerical study of blood flow in coronary artery bypass graft side-to-side anastomoses. Ann Biomed Eng.

